# Automated Three-Dimensional Detection and Shape Classification of Dendritic Spines from Fluorescence Microscopy Images

**DOI:** 10.1371/journal.pone.0001997

**Published:** 2008-04-23

**Authors:** Alfredo Rodriguez, Douglas B. Ehlenberger, Dara L. Dickstein, Patrick R. Hof, Susan L. Wearne

**Affiliations:** 1 Department of Neuroscience, Mount Sinai School of Medicine, New York, New York, United States of America; 2 Laboratory of Biomathematics, Mount Sinai School of Medicine, New York, New York, United States of America; 3 Computational Neurobiology and Imaging Center, Mount Sinai School of Medicine, New York, New York, United States of America; Harvard University, United States of America

## Abstract

A fundamental challenge in understanding how dendritic spine morphology controls learning and memory has been quantifying three-dimensional (3D) spine shapes with sufficient precision to distinguish morphologic types, and sufficient throughput for robust statistical analysis. The necessity to analyze large volumetric data sets accurately, efficiently, and in true 3D has been a major bottleneck in deriving reliable relationships between altered neuronal function and changes in spine morphology. We introduce a novel system for automated detection, shape analysis and classification of dendritic spines from laser scanning microscopy (LSM) images that directly addresses these limitations. The system is more accurate, and at least an order of magnitude faster, than existing technologies. By operating fully in 3D the algorithm resolves spines that are undetectable with standard two-dimensional (2D) tools. Adaptive local thresholding, voxel clustering and Rayburst Sampling generate a profile of diameter estimates used to classify spines into morphologic types, while minimizing optical smear and quantization artifacts. The technique opens new horizons on the objective evaluation of spine changes with synaptic plasticity, normal development and aging, and with neurodegenerative disorders that impair cognitive function.

## Introduction

Because of their capacity for plasticity and their key role as the location of excitatory synapses in the cerebral cortex, accurately characterizing the structure of dendritic spines is of profound biological significance. Spine morphology determines the strength, stability and function of excitatory synaptic connections that subserve the neuronal networks underlying cognitive function. Precise quantification of spine morphology in three dimensions (3D) is essential to understanding the structural determinants of normal neuronal function, its development, plasticity, and its dysfunction in neurodegenerative disorders. To date however, this level of precision has been restricted to time-intensive electron microscopy (EM) approaches with limited throughput, that are impractical for comparative population studies. In this paper we introduce a novel computational approach for detection and shape analysis of neuronal dendritic spines from confocal and multiphoton laser scanning microscopy (CLSM and MPLSM) images, that operates fully in 3D, and is faster and more accurate than existing semi-automated technologies. The algorithm is a module of our NeuronStudio software application [1], an integrated system for semi-automated digitization, morphometry and analysis of complex neuronal morphology at high resolution.

Spines come in multiple shapes and sizes, which subserve a diversity of function [2]–[6]. Although spine shapes in fixed tissue form a continuum rather than distinct categories, broad subclasses (e.g., thin, stubby, mushroom) have traditionally been distinguished on the basis of morphology (see refs [3], [7] for review). Spine morphology also varies dynamically in response to synaptic activity [8]–[13]. Smaller spines are less stable and more motile [14]–[16], and as a result, more plastic than large spines [17]. Recent data indicate that the size and morphology of the spine head are correlated with numbers of docked presynaptic vesicles [18], numbers of postsynaptic receptors [19], and hence with synaptic strength. From a biophysical viewpoint, these effects give rise to increased synaptic currents and reduced time constants for calcium compartmentalization in larger spine heads (see refs [5], [20] for review), modulating postsynaptic mechanisms that determine functions such as learning and memory [21]–[24]. Spine neck length and diameter also affects diffusional coupling between dendrite and spine [25]–[28], and spine density and shape regulate the degree of anomalous diffusion of chemical signals within the dendrite [29]. As more precise data from these studies emerges, the need for accurate spine morphometry in true 3D, and on large enough scales for robust statistical analyses becomes increasingly critical.

Digital representation of neuronal morphology using light microscopy has traditionally relied on manual tracing from a computer screen [30], [31], and is prone to subjective errors. Despite the recent introduction of semi-automated tracing methods (e.g., AutoNeuron, MBF Bioscience, Williston, VT), the problem of detecting and characterizing spine shapes automatically, in 3D, remains unsolved. In existing image analysis-based tools, precision at the finest scales is limited by the skeletonization methods used and by quantization errors. Even when imaged at the limits of CLSM resolution, single spines may span as few as 3–10 voxels, and neck diameters may be subvoxel resolution. Accurate recovery of spine geometry thus requires new analysis algorithms capable of using subvoxel information, and the full three dimensions of structural information from a LSM image stack. The spine detection and analysis method presented here is compatible with many methods for tracing the dendritic tree and thus can be implemented as an add-on module to existing manual or automated tracing packages. Spine voxels are clustered based on connectivity, analyzed with subvoxel precision using Rayburst-based shape analysis routines [1], and classified into three morphologic types, mushroom, stubby and thin. Procedures for declumping of merged spines and for combining detached spine heads with their stems are implemented to ensure accurate spine counts. The method is at least an order of magnitude faster than previous algorithms, and as a 64-bit application, can handle multi-gigabyte datasets. These algorithms have been tested and validated in our freely distributed software application, NeuronStudio [1], [32] (http://www.mssm.edu/cnic), which provides visual verification of spine classification and the ability for manual spine editing through interactive 2D and 3D displays.

## Results

### Image Segmentation and Voxel Processing

**Figure 1:**
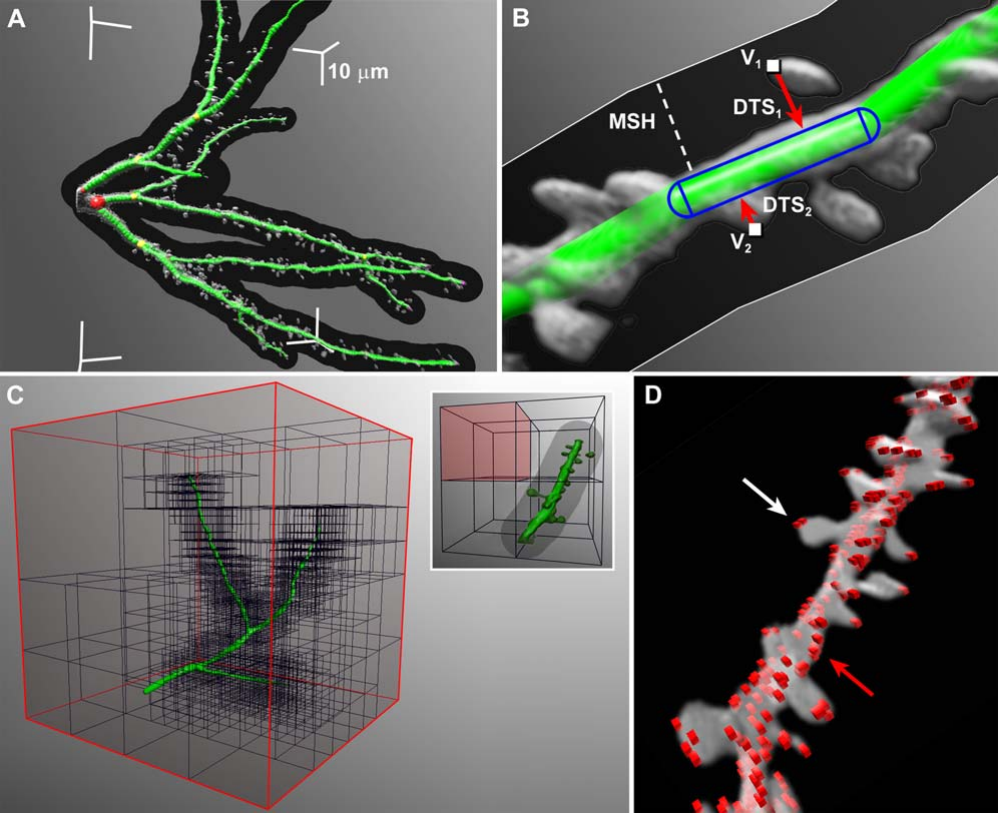
Processing of Candidate Voxels for Spine Detection. A) Dendritic model rendered as green balls superimposed on volume-rendered data (light grey voxels), augmented by the maximum spine height (*MSH*, dark grey envelope surrounding dendrites that contains all spine candidate voxels). Four corners of the axis-aligned bounding box (AABB) are shown, with the length of each axis segment representing 10 µm to depict the scale in 3D. B) Close-up view of a spiny dendritic section, volume rendered in grayscale, with superimposed 3D model formed by sequential green frustra. A single capsule formed by a cylindrical dendritic segment capped by two hemispheres at consecutive nodes is outlined in blue. Spine candidate voxels V_1_ and V_2_ are shown with their corresponding distance to surface (*DTS*) values (red arrows). The dark grey envelope around the dendrite represents the *MSH*, measured from the surface of the 3D model, as indicated by the white dashed line. C) Octree calculated for the top fork of the dendritic section shown in (A). The root node is outlined in red. Recursive subdivision of the 3D space results in increasingly smaller cubes surrounding the model. The inset on the right illustrates bulk rejection of voxels in a single leaf (red shaded cube) that does not intersect the *MSH* zone (grey envelope surrounding model) created by a small dendritic section. D) Volume-rendered spiny dendrite with voxels representing exterior maxima drawn as red cubes (voxel size exaggerated to enhance visibility). The number of exterior maxima is usually much greater than the number of spines, since multiple maxima may occur on a single spine (white arrow), or along the surface of the dendrites (red arrow).

The spine analysis module described here utilizes a previously computed model of the dendritic tree, comprising a series of nodes of specified diameter forming conical frusta (Fig. 1A,B), which could be obtained by any of several existing methods [33]–[37], and which is compatible with many existing neuronal morphometry applications [32], [38]. Because fluorescence intensity can vary with adequacy of filling, imaging depth and XY spatial extent in CLSM and MPLSM image stacks, segmentation based on a globally selected intensity threshold is in general not feasible, and requires a dynamically adjusted, local threshold.

Although the spine analysis method presented here is technically independent of the segmentation technique used, its performance will be enhanced as the efficacy of segmentation increases. In the case of threshold-based segmentation, faint or very thin spines may appear too small or be missed completely if the chosen threshold is too high. Likewise, too low a threshold will cause background noise to be segmented as part of the object, while spine shapes may appear larger, and distorted. For the current implementation we use an adaptation of the ISODATA method [39] to compute a local threshold at each node along the dendritic model. This method is appropriate for datasets exhibiting a bimodal distribution of intensity values, such as the grayscale images characteristic of deconvolved LSM image stacks, placing the threshold midway between the centroids of the two peaks of the distribution. To ensure an adequate sample of spine and background voxels for the ISODATA distributions regardless of the orientation of the dendrite, at each node along the dendritic model we define a cubic section of data centered at the node and having X, Y, and Z dimensions equal to 2.5 times the node's diameter. Voxels inside that domain are tested for intersection with the dendritic model, and only the intensities of voxels that do not intersect the model are used to compute the ISODATA threshold. It should be noted that excluding voxels based on intersection with the model is a non-trivial computation, requiring optimization in order to avoid significant overhead.

Once each node has been assigned a threshold value, any voxel in the dataset may be segmented by linearly interpolating a local threshold value between nodes along the closest dendritic segment. We use the term *spine candidates* for voxels with intensity values at or above their local threshold value, whose distance to the closest point on the surface of the model (*distance to surface*: *DTS*) is less than or equal to a user-defined parameter, the *maximum spine height* (*MSH*) expected for the dataset. Figure 1B shows *DTS* values (red arrows) measured for two sample spine candidate voxels in the data (*V_1_* and *V_2_*).

### Octree Partitioning to Optimize Voxel Processing

Neurons are sparsely distributed within an image volume and spine candidate voxels occupy only a small fraction of the dataset. To identify and process spine candidate voxels efficiently we utilize a data structure known as an octree [40], which recursively organizes objects distributed inside a 3D volume, allowing fast searching by spatial location (Fig. 1C). We first define an axis-aligned bounding box (AABB) around the entire dendritic model, which becomes the root of the octree structure (outer cube drawn in red, Fig. 1C), and contains references to all dendritic segments. An AABB is a bounding volume used to optimize the computation of intersection tests in 3D. It comprises a rectangular box having faces aligned with the major axes rather than with the geometry of the object (Wikipedia article at: http://en.wikipedia.org/wiki/Bounding_volume). The root AABB is subsequently partitioned into eight octants that are each further subdivided, recursively (inset, Fig. 1C). Each octant becomes a child of its parent in the resulting octree structure. At successive subdivisions, the dendritic segments in the parent are tested for intersection with each of its children. Each child then receives a reference only to those dendritic segments that intersect it, and therefore receives a subset of the segments in the parent. Children with less than a pre-specified number of references are not divided further. These are termed the leaves of the octree.

The octree improves execution in three ways: (i) by allowing bulk rejection of voxels whose *DTS* exceeds the *MSH* (e.g., the red shaded cube in inset, Fig. 1C); (ii) by allowing rapid identification of model segments within the neighborhood of each spine candidate voxel; (iii) by rapid rejection of voxels failing to meet a minimum threshold established by the dendritic segments intersecting that leaf.

Before partitioning, the radii of the model's dendritic segments are augmented by the *MSH*, creating an envelope around the dendrites that includes all candidate spine voxels (dark grey envelope surrounding the model, Fig. 1A–C). Sections of the imaged volume that do not intersect these augmented segments are not divided further and voxels inside these sections can be excluded from our list of candidates (inset, Fig. 1C). Without the octree optimizations, the computational expense of the algorithm would be O(*M*×*N*), where *M* is the number of voxels in the data, and *N* is the number of edges in the model. As an example, a dataset where the spine analysis took 22 seconds with the octree in place, required over five minutes to process with the octree disabled.

### Spine Detection Using Voxel Clustering

**Figure 2:**
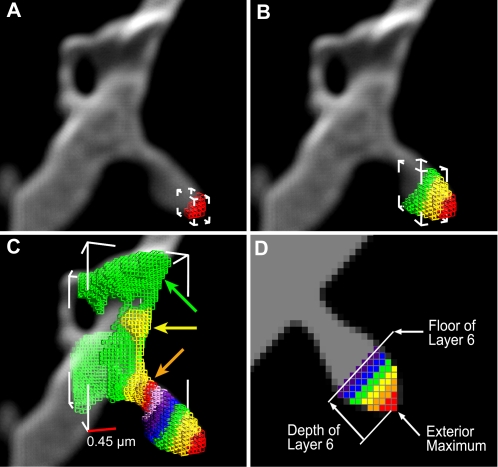
Cluster-Building by Iterative Addition of Layers. Individual voxels are rendered as wireframe cubes in successive layers of different colors, superimposed on a volume-rendered dendritic spine. A) Voxels of first layer shown in red. White frame shows each corner of the layer's AABB. B) Fourth iteration of the cluster-building algorithm produces the green layer, and correspondingly larger bounding box. C) In the last iteration, the diagonal of the bounding box exceeds the user-provided maximum spine width (*MSW*), as the green layer floods into the dendrite (green arrow). The yellow layer is the first to exhibit a spread ratio exceeding *SR*_(crit)_ (yellow arrow). Hence, the layer immediately preceding this becomes the spine base (orange layer, orange arrow). Scale bar shown as red horizontal edge of lower left corner of AABB represents 0.45 µm. D) Schematic of cluster layer-building, showing exterior maximum (red cube at maximum point); floor of layer 6 (proximal end of purple layer) and depth of layer 6 (distance from exterior maximum to floor).

Individual spines are detected by clustering candidate spine voxels, starting from the tips and moving towards the dendrite. Voxels lacking 26-connected candidate neighbors with a greater *DTS* value are termed *exterior maxima*, and represent local high points that can occur at the tips of spines as well as surface irregularities and imaging noise along the surface of the dendrite (red voxels in Fig. 1D). Exterior maxima are processed in decreasing *DTS* order to ensure that each cluster uses the highest maximum. The cluster-building algorithm can be described as an iterative 3D flood-fill of the structure starting from an exterior maximum. Each iteration builds a layer by establishing a floor value that limits cluster growth towards the dendritic segment but does not constrain sideways growth for that iteration (Fig. 2). Successive layers are created by adding all 26-connected neighbors of the previous layer, and establishing the minimum *DTS* of these voxels as the floor value for the layer. All unvisited voxels connected to the current layer with a *DTS* greater than or equal to the floor value, are then added in a number of subiterations. For the first layer, the exterior maximum and its immediate neighbors establish its floor. Pseudo-code implementing an optimized version of the cluster building algorithm is provided in Box S1 (Supporting Information, online).

As voxels are added to individual layers, we maintain an AABB for the layer by updating the minimum and maximum voxel coordinates in all three dimensions (white bounding box, Fig. 2A–C). We estimate the size of the layer continuously as voxels are added by computing the diagonal of the bounding box, which we term the *spread of the layer*. If at any point during layer-building the spread exceeds a user-provided *maximum spine width*, the cluster building stops (see green layer flooding into dendrite, Fig. 2C).

### Calculating Spine Profiles

For each layer built in a particular cluster, we maintain a profile of measures for later use by the shape classification routine. We first compute the center of mass of each layer and run the 2D variant of our Rayburst Sampling algorithm [1] from that center of mass to estimate the diameter at that layer. The *Rayburst diameter* is the diameter obtained by measuring the minimum surface-to-surface span inside a tubular structure (see Rodriguez et al. [1] for full mathematical details). For structures assumed to have an approximately radially symmetric cross section, 2D Rayburst run in the XY plane is insensitive to residual Z axis smear from incomplete deconvolution, yielding a reliable estimate of the structure's diameter irrespective of its orientation within the image stack. The spine profile contains three values: the *spread of the layer*; the *Rayburst diameter*, as defined above, and the *depth of the layer*, computed as the distance from the exterior maximum to the floor of that layer (Fig. 2D). The base of the spine is the last layer in the cluster that should be considered part of the spine. For detached clusters the algorithm produces a final layer containing no voxels. This layer is always considered the base and its depth is set to the *DTS* of the maximum, since no floor was defined. To determine the base of an attached cluster we search its layers sequentially, from first to last, for the first layer that displays a sudden increase in spread (e.g., Fig. 2C, yellow arrow, yellow layer). The base is the layer immediately preceding this (e.g., Fig. 2C, orange arrow, orange layer). We quantify this spread increase, for each layer *i* in a cluster, in terms of the *spread ratio* (*SR*), given by the expression:
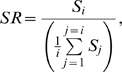
(1)where *S_i_* is the length of the diagonal of the AABB encompassing all voxels in the *i*th (current) layer; *S_j_* is the length of the diagonal of the AABB encompassing all voxels in the *j*th layer, for all *j*≤*i*. The layer preceding the first layer encountered with an *SR* value greater than a critical value *SR_(crit)_* is selected as the base. An empirically determined value of *SR*_(crit)_ = 1.5 was optimal for the data analyzed in the present study. All layers in the cluster after the base are then discarded, and their voxels may later enter into another cluster. For attached clusters, the last layer has a spread larger that the maximum spine width allowed (final green layer, Fig. 2C), therefore the spread and Rayburst diameter are set to infinity to ensure that this layer is never selected as the base. Once the base layer is identified, we compute the aspect ratio (*AR*) of the cluster, defined as the ratio of the depth to the spread of the base layer (Fig. 2D). Clusters with low aspect ratio values (less than 0.25 in our empirical datasets) represent dendritic surface irregularities that are too flat to be considered spines, and can be safely discarded. Clusters may also be discarded based on user-supplied parameters for minimum voxel count and minimum spine height (depth of base layer). All remaining clusters are classified as spines.

### Cluster Declumping

**Figure 3:**
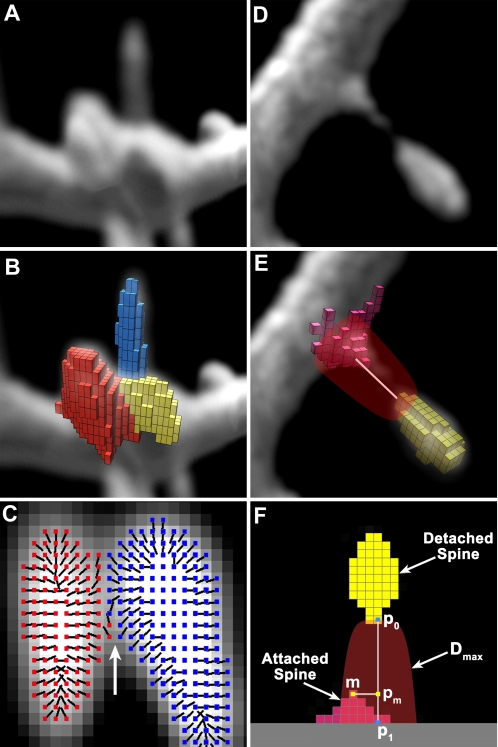
Cluster Declumping and Spine Stem Reattachment. A) Volumetric data showing cluster of three spines that appear merged due to limited image resolution. B) Result of clustering algorithm using the cluster declumping routine described in the text. The merged spines have been properly detected as separate clusters indicated by differently colored voxels. C) Local 3D gradient vectors used to declump two merged spines. Vector heads are colored red or blue; black tails point in direction of increasing intensity. At the valley marked by the white arrow, the gradients reverse direction: red gradients point leftward, toward the center of mass of the red spine; blue gradients point rightward, toward the center of mass of the blue spine. D) Volumetric data showing an apparently detached spine head, and its spine stem attached to the dendrite. Because of poor resolution of the spine neck, this thin spine is detected as two separate spines. E) Bell-shaped region (transparent red) used to detect the stem of a spine whose neck cannot be adequately resolved. The voxels of the attached (red) and detached (yellow) spines are represented as cubes within a volume rendering of the dataset. Because the tip of the attached spine falls within the bell-shaped region, the algorithm merges these two clusters into a single spine. White line between yellow detached and red attached spines represents an approximate scale of 0.8 µm. F) Schematic showing parameters of the spine stem reattachment routine. *p_0_*: lowest-*DTS* voxel on a detached spine; *p_1_*: closest point to surface of the dendritic model: *m*: maximum point on an attached spine within the bell-shaped domain shown in transparent red; *p_m_*: projection of *m* onto line segment [*p_0_*, *p_1_*]; *D_max_*_:_: limit of the bell-shaped domain that encloses the spine stem.

Cluster shape is calculated in 3D and used both to remove non-spine clusters and to classify spines into types. Inadequate image resolution, inaccurate thresholding, or sheer physical proximity can cause adjacent spines to appear merged (e.g., Fig. 3A). We introduce a declumping method that effectively separates merged spines using intensity gradient information. In spiny dendrites imaged with CLSM or MPLSM, apparently merged spines are relatively common, making cluster declumping an essential precursor to spine shape analysis (Fig. 3B). When two spines are in contact, layers built from the exterior maximum of the first spine may extend into the adjoining spine. Voxel intensities are naturally brighter at the center of spines and dimmer at the edges, forming an intensity landscape that can be used to delimit individual hills (spines) and valleys (edges) (Fig. 3C). Declumping uses intensity gradients, computed as 3D vectors at each spine voxel, to detect the brighter centers during the layer building (red and blue vectors, Fig. 3C). As each layer is built, the center of mass of the voxels used to establish the layer's floor is calculated. For the first layer, we compute the center of mass of the exterior maximum and its immediate neighbors. We then define a *layer attachment line* from this center of mass to the closest point along the medial axis of the dendritic tree. To prevent the layer from crossing an intensity valley into an adjacent spine, only those voxels whose intensity gradient points towards the layer attachment line are considered for inclusion in the spine layer.

### Spine Shape Classification

**Figure 4:**
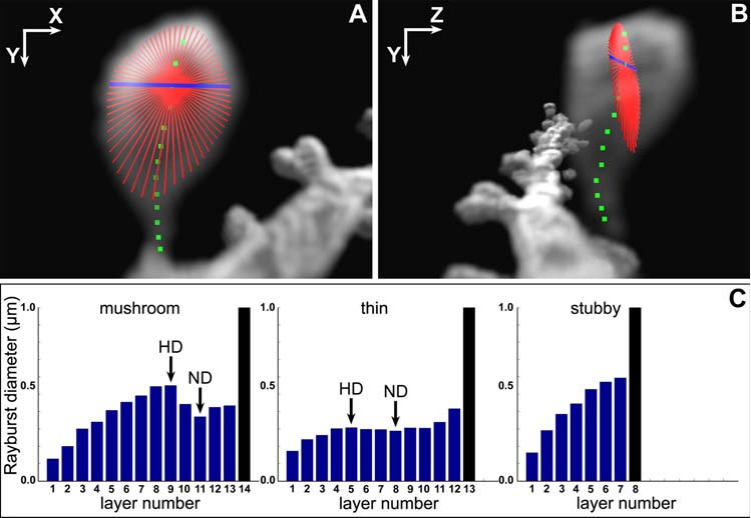
Shape Classification Using Rayburst Diameters of Consecutive Layers. A) XY view showing the rays (red lines) of a 2D Rayburst run at the center of mass (green square) of a single layer. The thick blue line indicates the resulting width of the structure as calculated by Rayburst, and provides an approximate scale of 0.7 µm. B) Side profile of the Rayburst core, demonstrating how the rays extend in the XY plane only, avoiding the effects of optical smearing in the Z direction. C) Bar graphs showing the blue Rayburst diameters calculated at each layer as a function of the layer number, for three representative spines of type, mushroom, thin and stubby. These profiles of Rayburst diameters are used to classify spine types. *HD*: head diameter; *ND*: neck diameter. For attached spines, the last layer's Rayburst diameter (black bar) is set to infinity.

**Figure 5:**
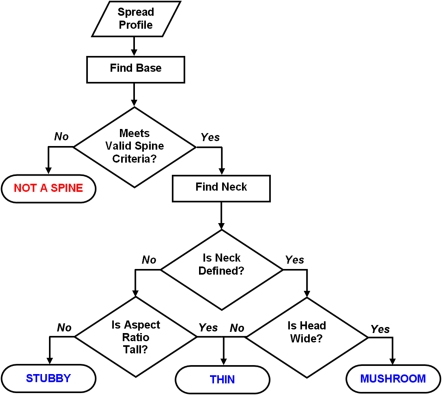
Flowchart of Algorithm Used for Spine Classification. The spread profile of the cluster is used to determine the base of the spine. From this computation an aspect ratio (*AR*) is calculated for the cluster to determine whether a spine has been found. For valid spines, the presence of a neck indicates a mushroom or thin spine, whereas absence of a neck indicates a stubby or thin spine. The aspect ratio and the width of the head (head diameter; *HD*) are used to resolve the final spine types.

After clustering, spine shapes are classified into three types, mushroom, stubby and thin, using the profile of 2D Rayburst diameters computed in consecutive layers along the length of the spine. Use of 2D Rayburst within each layer avoids the effects of residual optical smearing in the Z direction that arise from incomplete deconvolution [1], providing a reliable, high resolution profile of spine shape. Figure 4A,B shows a 2D Rayburst run from the center of mass of the third layer in a typical mushroom spine. The blue horizontal lines superimposed on the red Rayburst vectors in the XY view (Fig. 4A) and ZY view (Fig. 4B) indicate the resulting diameter in layer 3. Consecutive Rayburst diameters are plotted in Figure 4C versus layer number as bar graphs. These profiles of Rayburst diameters are used to classify spine shapes. Spine shape classification is controlled by critical values of the following three parameters: *AR* (defined in *Calculating Spine Profiles*), head to neck ratio (*HNR*) and head diameter (*HD*). These three parameters, together with the *spread ratio* defined in Equation (1), control the decision tree of Figure 5 that classifies spine shapes. First the s*pread ratio* is used to determine the base of the spine. Then the *AR* is computed, and clusters are separated into valid spines (those that are sufficiently tall and narrow; *AR*>*AR_spine*_(*crit*)_) or invalid spines (those that are too wide and flat). For valid spines, existence of a neck is the first decision point in the scheme. The measurements *HNR*, *neck_position* and *head_position* are returned by the following algorithm, which iterates sequentially through the layers of the spine profile:

**SET** HNR to 0

**SET** neck_position to 0

**SET** head_position to 0

**FOR** every layer i in the profile

**FOR** every layer j < i

**SET** r to rayburst_diameter[j] / rayburst_diameter[i]

**IF** r > HNR **THEN**

**SET** HNR to r

**SET** neck_position to i

**SET** head_position to j

**END IF**

**END FOR**

**END FOR**

Spines with *HNR* greater than a critical value, *HNR*_(*crit*)_, are considered to have a neck. For detached spines, the above algorithm is not needed since we simply select the last layer as the neck, and the largest layer before the neck as the head. Spines with a neck can be either thin or mushroom types. If any layer above the neck has a Rayburst diameter exceeding a critical value (*HD*_(crit)_), the spine is classified as a mushroom, otherwise it is a thin (see flowchart, Fig. 5). For spines lacking significant necks, an aspect ratio less than *AR_thin*_(*crit*)_ indicates a stubby, otherwise the spine is labeled as thin. By optimizing against manually classified data, we empirically determined *AR_thin*_(*crit*)_ to be 2.5; *HNR*_(*crit*)_ to be 1.1 and *HD*_(*crit*)_ to be 0.35 µm for the data analyzed in this study.

### Spine Stem Reattachment

Extremely thin necks that occur on some spines cannot be resolved under LSM, leading to apparently detached spine heads (e.g., Fig. 3D). The end of the neck closest to the dendrite, termed the *spine stem*, may protrude far enough for it to be detected as a cluster and classified as a separate spine (red voxels, Fig. 3E), artificially increasing spine counts. Before spine counting and classification, we search for potential spine stems by determining if any attached spines without necks are located directly below a detached spine. Given the location of the lowest-*DTS* voxel in a detached spine, *p_0_*, we compute the closest point on the surface of the dendritic model, *p_1_*, (Fig. 3F). These two points define a line, *L*. For any attached spine in a prespecified neighborhood of *p_0_*, we project its maximum voxel, *m*, onto *L*. The attached spine is identified as the stem if the projection point, *p_m_*, lies within the line segment [*p_0_*, *p*_1_], and its perpendicular distance to the line is less than the value *D*_max_ given by the expression for a bell-shaped domain (Fig. 3F):
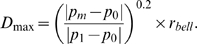
(2)Here *r_bell_* represents the maximum radius at the base of a bell-shaped domain around the line segment [*p*_0_, *p*_1_] in micrometers, obtained empirically for our data. If more than one attached spine meets these criteria, the spine with the smallest perpendicular distance is chosen as the stem, and the two clusters are combined into a single spine.

### Validation of Automated Spine Detection and Shape Classification

**Figure 6:**
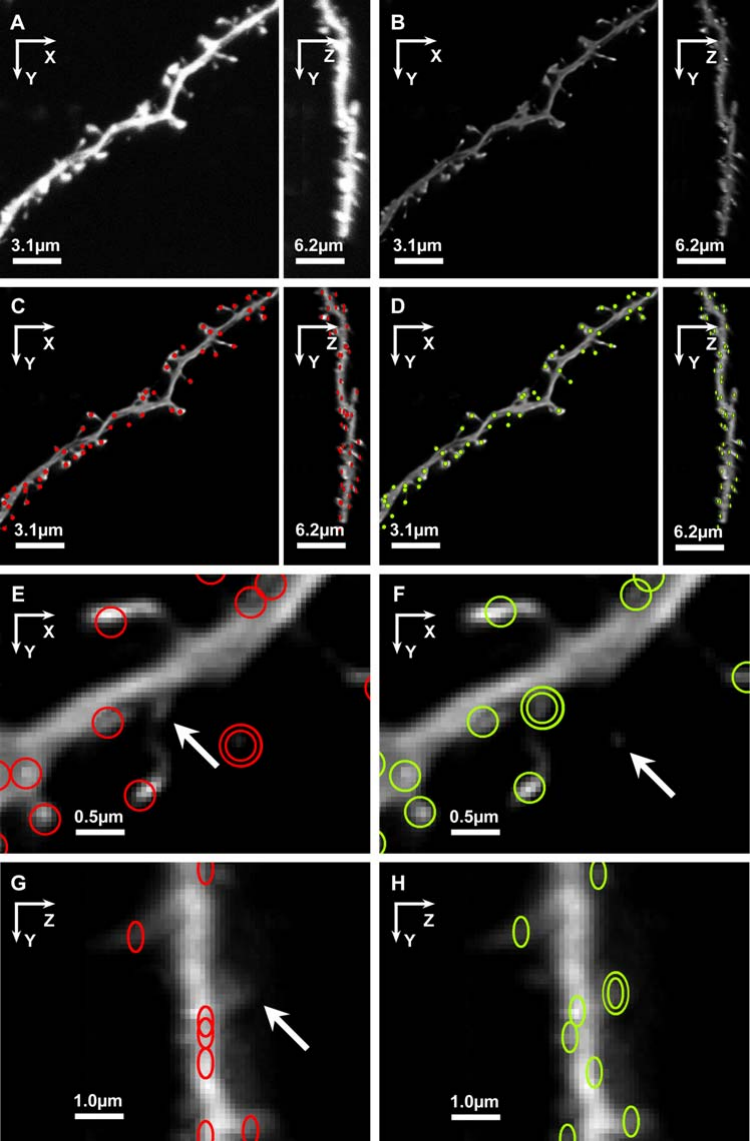
Manual and Automated Results for Typical Image Data. A,B) XY and ZY maximal projections of half the field of view of a typical image stack before (A) and after (B) deconvolution. Compared to the raw data (A), the deconvolved data exhibit good relative intensity equalization of spines and dendrites, and significantly reduced Z-axis “stretching” from optical smear, in the ZY projection (B). Note the difference in scale in XY vs ZY projections, reflecting the fact that voxel dimensions are twice as big in Z as in X and Y; (voxels are [0.05 µm, 0.05 µm, 0.1 µm]). C, D) Comparison of spines detected by one of the manual operators (red circles, C) and automatically by NeuronStudio (green circles, D). E,F) Close-up XY view of a spiny branch comparing spines detected manually (red circles, E) and automatically (green circles, D). Examples of typical mismatches between the manual and automatic detection methods are highlighted with double circles (double red circles: Manual-only detection; double green circles: *NS-only* detection), and by white arrows (misses). The short, stubby spine projecting downward from the dendrite is missed by the manual operator (white arrow, E), but detected by NeuronStudio (double green circles, F) A very faint, thin spine that is missed by NeuronStudio (white arrow, F) is detected by the manual operator only (double red circles, E). G,H) Close-up ZY view of a spiny branch comparing manual with automated spine detection. Example of a stubby spine projecting in the Z direction, that is typically missed by the manual operator (white arrow, G) but detected by *NS-only* (double green circles, H).

We validated the detection and classification algorithms by comparison with trained human operators on the same data. Seven CLSM image stacks, each containing at least one spiny dendritic segment such as that shown in Figure 6, were collected according to the procedure described in Methods. NeuronStudio was used to detect and classify spines automatically in these data, and the results were compared to those obtained independently by four skilled operators using manual methods. Because the problem of classifying spine shapes is independent of the detection method used, we evaluate these two processes separately. Figure 6 shows XY and ZY maximal projections of approximately half the field of view of a typical image stack. Before deconvolution (Fig. 6A), the spines are visible in XY, but substantially obscured by the brighter dendrite in the ZY view. Following deconvolution (Fig. 6B), relative intensities of spines and dendrite are closer, and individual spines are clearly discernible in the ZY projection (Fig. 6B).

### Comparison of Automated and Manual Spine Detection

A skilled operator examined each of the confocal image stacks interactively, first using a 2D slice viewer and then a 3D volume rendering. In the manual procedure, the operator labeled each identified spine with a marker labeled ‘manual’. NeuronStudio then identified spines in the same dataset automatically, placing a marker labeled ‘auto’ at the center of mass of each spine. Automatically and manually detected spines were compared on a spine-by-spine basis and the counts for both methods, for the manual method only, or for NeuronStudio only, were tabulated for each stack (Table 1). Across the seven image stacks, the manual operator detected 447 spines, and NeuronStudio detected 492. Of these 492, 401 were detected by both the manual and automated methods.

**Table 1:** Validation of Automated Spine Detection.

Stack	Manual	NeuronStudio	Both	NS-only	Manual-only
2	70	71	65	6	5
4	68	77	56	21	12
6	58	67	57	10	1
8	79	89	70	19	10
9	59	64	54	10	5
G	58	67	51	16	7
K	55	57	48	9	8
Total	447	492	401	91	48

From left to right, columns indicate: *Stack*: stack identifier; *Manual*: numbers of spines in each stack detected by the manual method; *NeuronStudio*: numbers of spines in each stack detected by NeuronStudio; *Both*: spines detected by both methods; *NS-only*: spines detected by NeuronStudio only; *Manual-only*: spines detected by the manual method only.

Spines detected by both manual and automated methods were considered correct hits. Spines detected by the manual method only (*Manual-only*, Table 1), or by NeuronStudio only (*NS-only*, Table 1), were potential errors. Those detected by *NS-only* could be either false positives, or correct detections that were missed by the human operator. Examples of spines detected manually (red circles), by NeuronStudio (green circles), *Manual-only* (double red circles) and *NS-only* (double green circles) are shown in XY and ZY projections of typical image stacks, in Figure 6. *NS-only* and *Manual-only* spines were subsequently analyzed by another three independent human operators. This showed that most *NS-only* spines were small, flat stubby types, or short thin types, projecting primarily above and below the dendrite, along the Z direction where they are more difficult for a human operator to distinguish (e.g., white arrow, Fig. 6G and double green circles, Fig. 6H). A small percentage of *NS-only* spines were judged actual false positives – dendritic surface bumps too small to be considered actual spines or non-reattached spine stems. *Manual-only* spines represent potential misses by NeuronStudio. Subsequent examination of M*anual-only* spines showed that about half were from clusters of two or three adjacent spines that did not declump completely. Of the remainder, about half were very dim voxel clusters, below the local threshold established by NeuronStudio, but just detectable by the human operator (e.g., double red circle, Fig. 6E and white arrow, Fig. 6F), while the rest were judged actual false positives.

### Comparison of Automated and Manual Spine Shape Classification

Following detection, spine shapes were classified by NeuronStudio. Three skilled human operators (A, B, and C) then examined the same set of spines in 3D with default markers superimposed on the volume rendered data, using NeuronStudio interactively to rotate, zoom, and inspect the data from any angle. The three operators classified each spine independently by setting the default marker type to ‘stubby’, ‘thin’, or ‘mushroom’, according to the observed spine shape and a set of previously agreed classification criteria. A *human consensus* (HC) classification was also established as the type designated by two or more of the three human operators for each spine. To assess intra-operator variability, one operator (Operator C) performed the classification twice, evaluating the same datasets on different days. After adjusting NeuronStudio's shape classification parameters to fit the HC, spine types were classified automatically. Table 2 shows the type counts for each operator, and the HC and NeuronStudio classifications. For each spine type, variability between operators was evaluated by measuring the percent match between each pair of operators on a spine-by-spine basis. All pairwise percent matches on the same 442 detected spines are shown in Table 3 (higher percent match indicates lower variability between operators).

**Table 2:** Table of Spine Classification Counts by Morphologic Type.

	Operator A	Operator B	Operator C_1_	HC	NS (trained to HC)	Operator C_2_
**Stubby**	113	112	154	122	114	130
**Thin**	257	250	210	241	259	235
**Mushroom**	60	69	66	67	65	66

Comparison of numbers of spines in each of the three types, classified by the automated method (NeuronStudio: NS) and manual methods (human operators A, B, C). To assess intra-operator variability, Operator C performed the same task on two different days: C_1_ and C_2_. HC: human consensus, the spine type designated by at least two of the three human operators. NS (trained to HC): Spine counts for each type classified automatically by NeuronStudio after the shape parameters of the decision tree, Figure 5, were optimized to best match the HC.

**Table 3:** Intra- and Inter-Operator Percent Match in Shape Classification by Morphologic Type.

	Intra-Operator Percent Match	Inter-Operator Percent Match			
Comparison	C_1_–C_2_	A–B	C_1_-A	C_1_-B	NS-HC
**Stubby**	78.6%	82.3%	90.3%	90.2%	82.8%
**Thin**	91.0%	88.7%	77.0%	78.0%	92.1%
**Mushroom**	74.2%	90.0%	70.0%	68.1%	79.1%
**Overall**	82.9%	85.8%	79.0%	78.3%	85.8%

Comparison of intra-operator (C_1_–C_2_) and inter-operator variability between human operators (A–B; C_1_-A; C_1_-B) and between the automated (NeuronStudio) spine classification and the human consensus (NS-HC). Variability is measured by pairwise percent match: higher percent match indicates lower variability between operators. Overall, NeuronStudio's classification matched the HC standard equally, or better than, the best human inter-operator match.

Because many spine types are ambiguous, shape classification by human operators is subjective and prone to high variability both within an operator on the same spines (intra-operator variability), and between trained operators using the same classification criteria (inter-operator variability). Pairwise percent match between operators ranged from 78.3% to 85.8% (Table 3). Percent match within human operator C on two different days was 82.9% (C_1_–C_2_, Table 3). NeuronStudio's classification matched the HC classification 85.8% of the time after adjusting the shape parameters (Table 3). Within each spine class, NeuronStudio matched 79.1% of mushroom spines; 82.8% of stubby and 92.1% of thin spines classified by the human consensus. In general NeuronStudio matched the HC standard equally, or better than, the best inter-human match rates (Operator A to Operator B, 85.8% overall), and better than the intra-operator match (Operator C_1_–C_2_, 82.9% overall, Table 3). Automated classification by NeuronStudio has the advantage of removing human subjectivity and intra-operator variability, and the parameters can be optimized to match a particular classification criterion.

### Symmetry of Detection and Classification

**Figure 7:**
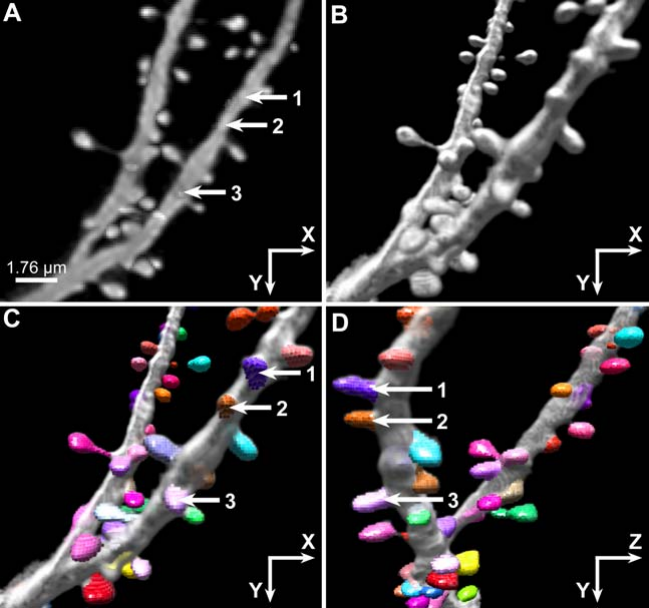
3D Information is Required for Accurate Spine Detection. Two- and 3D renderings of a spiny dendrite imaged with CLSM and reconstructed with NeuronStudio with results of spine detection algorithm. A) Image stack is shown as a 2D maximal projection with arrows indicating positions of three representative spines (numbered 1,2,3) oriented perpendicular to the image plane, that cannot be detected from a 2D projection. B) The same dataset shown as a 3D volume rendering. The three spines are somewhat more visible in the 3D XY view. C) XY view of spines detected in 3D by NeuronStudio with spine voxels represented in different colors, superimposed on volume rendered data. Arrows point to spines 1, 2, and 3, not visible in 2D, that can be detected by NeuronStudio in 3D. D) ZY view of the same dataset as shown in (C), rotated through 90° to show the prominence of these spines in 3D.

To evaluate the algorithm's performance on spines of different orientations with respect to the image plane and around the dendritic structure, for each spine we record the angle formed by the spine's primary axis with the image plane. Spines were divided into two groups based on this angle: *mostly horizontal*, those with absolute values of angle≤45 degrees from the image plane, and *mostly vertical*, those with absolute values of angles>45 degrees from the plane. For dendrites running approximately parallel with the image plane, the *mostly horizontal* spines are visible on the sides of the dendrite, in the XY view (e.g., Fig. 6E,F and Fig. 7A,B,C). The *mostly vertical* spines tend to project orthogonal to the image plane, being more visible in the ZX and ZY views (e.g., Fig. 6G,H and spines marked 1,2, and 3 in Fig. 7D).

### Symmetry of Spine Detection

To evaluate the symmetry of detection for all orientations, we compared the number of spines detected by the automated and manual methods in the *mostly horizontal* and *mostly vertical* groups. The results are shown in Table 4. Both the manual and automated methods reported more spines in the *mostly horizontal* orientation than the *mostly vertical* orientation (Table 4). This result confirms that when using light microscopy, spines with long axes pointing along the optic axis, and particularly those positioned directly above and below the dendrite are significantly less visible than those parallel with the image plane. This asymmetry was greater in the manual method (65.0% *mostly horizontal*:35.0% *mostly vertical*, Table 4) than the automated method (58.5% *mostly horizontal*:41.5% *mostly vertical*, Table 4), because the automated method was better at detecting sharp bumps projecting mostly vertically, above and below the dendrite, while the manual method was better at detecting very faint spines on the sides of the dendrite (see “Comparison of Automated and Manual Spine Detection”).

**Table 4:** Symmetry of Spine Detection with respect to Orientation.

		Mostly Horizontal		Mostly Vertical	
	Total Detected	Spine Count	Percent of Total	Spine Count	Percent of Total
**Automated Detection**	491	287	58.5%	204	41.5%
**Manual Detection**	449	292	65.0%	157	35.0%

Spines detected according to orientation with respect to the XY plane, for automated versus manual methods. The first data column shows the total number of spines detected for each method. The second and third columns show the number of spines detected in the *mostly horizontal* orientation, and those numbers expressed as percentages of the total detected, respectively. The fourth and fifth columns show the number of spines of each type detected in the *mostly vertical* orientation, and those numbers expressed as percentages of the total. Both automated and manual methods tend to detect more spines in the *mostly horizontal* orientation, with slightly higher horizontal-to-vertical asymmetry in the manual method than the automated method.

### Symmetry of Spine Classification

To evaluate the symmetry of spine shape classification, we compared the type composition (measured as percentages of mushroom, stubby and thin types relative to total spine numbers) of the automated and manual methods in the *mostly horizontal* and *mostly vertical* groups. The results are summarized in Table 5. Spine type compositions were very similar in the *mostly horizontal* and *mostly vertical* orientations, for both manual and automated classification methods. In each method, substantially fewer spines were classified as mushroom and thin types, and substantially more spines were classified as stubby, in the *mostly vertical* relative to the *mostly horizontal* group (Table 5). The alteration in type composition moving from *mostly horizontal* to *mostly vertical* orientations was very similar in the automated and manual classification methods. We infer from this that the altered type composition in the vertical orientation arose primarily from shape artifacts due to effects such as poorer resolution, and residual optical smear, in the Z direction. Future research will focus on developing methods to adjust shape classification parameters depending on spine orientation, and degree of residual optical smear in the images.

**Table 5:** Symmetry of Spine Classification with Respect to Orientation.

	Mostly Horizontal		Mostly Vertical	
	Automated Classification	Manual Classification	Automated Classification	Manual Classification
Mushroom	25.4%	21.4%	5.9%	8.0%
Stubby	9.4%	8.9%	58.8%	51.1%
Thin	65.2%	69.6%	35.3%	40.8%

Type composition of *mostly horizontal* and *mostly vertical* orientations for the automated and manual classification methods. Each column shows the percentage of spines classified as mushroom, stubby and thin for a given orientation, and method. The type compositions vary substantially with orientation: percentages of mushroom and thin spines are reduced, while percentages of stubby spines are increased, in the *mostly vertical*, relative to the *mostly horizontal* orientations. The type compositions do not vary substantially with classification method: manual and automated methods have similar type compositions within the mostly horizontal and mostly vertical orientations.

### Imaging Requirements and Algorithm Performance

For this study the data were imaged at a resolution of 0.05 µm in XY (lateral resolution) with 0.1 µm steps along the optical axis (axial resolution). We have found that the algorithm can successfully detect spines at resolutions as low as 0.2 µm in any direction. For reliable shape classification, we recommend that voxel resolution be maintained at 0.1 µm or higher in all directions. Whatever the chosen resolution, our method requires that the LSM data be properly deconvolved in order to reduce the optical smearing introduced by the point spread function (PSF) of the microscope as well as to filter out any shot noise created by the CCD camera during digitization.

We tested the algorithm's performance on a Windows workstation with an Intel Xeon 1.0 GHz processor and 1GB of RAM. The execution time for a representative image stack of size 512×512×100 at a resolution of 0.05×0.05×0.10 µm containing a single branch with about 70 spines was 12.8 s. When the algorithm was run on a composite of 7 stacks of the same resolution and similar spine distribution as above, the running time increased to 90 s, representing a linear increase in execution time with input size. Because some overhead is incurred in spine management, the observed linearity can be affected by the spine density in each dataset. In general the execution time should remain proportional to the number of voxels examined, which ultimately depends on the size of the dataset. Output of the spine analysis can be saved as a text file (see Table 6).

**Table 6:** Spine Analysis Output Format.

ID	SECTION NUMBER	SECTION LENGTH	X	Y	Z	HEAD DIAMETER	NECK DIAMETER	MAX-DTS	TYPE	ANGLE WITH XY PLANE
1	0	36.33	24.54	3.47	2.23	0.56	0.36	1.84	mushroom	53.76
2	0	36.33	11.73	13.70	5.03	0.17	0.005	1.52	thin	−38.41
3	0	36.33	17.58	7.77	3.70	0.26	N/A	1.49	thin	−15.86
4	0	36.33	5.06	17.91	6.79	0.33	N/A	1.18	stubby	−81.66
5	0	36.33	12.61	11.88	5.04	0.65	0.56	1.12	mushroom	3.60
6	0	36.33	7.59	15.43	5.01	0.27	N/A	1.11	stubby	−63.30

Representative section of the spine analysis output file. For each spine detected, the output file contains the following information: a numerical identifier, the identifier for the dendritic section (segment between successive branchpoints) where the spine is located, the length of the dendritic section, the physical coordinates of the spine's center of mass in the image stack, head and neck diameters (if applicable), the distance to surface for the tip of the spine, the type of the spine, and the angle of the primary spine axis with respect to the XY image plane.

## Discussion

The emerging appreciation within Neuroscience generally, that spine morphology is a sensitive index of functional and structural plasticity, has generated a rapidly increasing demand for tools that can reconstruct, classify and quantify spine shapes. The ability to analyze large volumetric data sets accurately, efficiently, and in true 3D has been a major bottleneck in deriving robust relationships between altered neuronal function and changes in spine morphology. Traditional computer-assisted manual methods for digitizing spines remain time-consuming, inaccurate and subjective (e.g., NeuroZoom [30], Neurolucida [31] [MBF Bioscience, Williston, VT]). Even with the advent of semi-automated tracing methods, characterization of fine dendritic and spine structures in true 3D remains a difficult challenge.

To the best of our knowledge, three methods for automated dendritic spine analysis from light microscopy images have been reported in the literature, only one of which operates on 3D data. This 3D method, by Koh et al. [41], uses the “grassfire” propagation technique for assigning each dendritic voxel a distance to the medial axis of the dendritic structure [42]. Tips and protrusions on the surface of the dendrite are identified as local maxima in this metric. While the method produces good results on a number of spine configurations, the iterative nature of the grassfire algorithm results in slow execution times on modestly sized datasets, on the order of half an hour for a 512×512×512 stack, the typical size to reconstruct a single spiny branch. The use of a global user-defined segmentation threshold further limits the applicability to small image stacks in which relatively uniform fluorescence levels can be attained.

More recently, Zhang et al. [43] have used a curvilinear structure detector to detect spines in 2D projections of LSM image stacks, and linear discriminant analysis to differentiate true spines from pseudospines. The method improves upon earlier techniques by using a local adaptive threshold for spine and dendrite detection, but is implemented only in 2D, from a maximal projection. In such 2D methods, spines above and below the dendrite projecting along the optic axis cannot be detected reliably, and spine shapes, lengths and other measurements are necessarily distorted by the absence of information in the Z direction. Nor is sufficient information available to discretize adjacent spines that appear merged in the 2D segmented images, artificially altering computed spine counts and densities. Recently, the same group has addressed some of the problems with the Koh method [41] by introducing various forms of adaptive thresholding and using a more efficient method of detecting spine tips [44]. This implementation, however, remains 2D-based and its application to spine counts and shape analysis is accordingly limited.

The automated spine detection and shape analysis algorithms presented in this paper directly address these limitations, providing significant advances over existing techniques. By operating on minimal subsets of voxels defined by the octree, our algorithm avoids many of the computational constraints encountered by previous spine analysis techniques [41], [43]. Although the current implementation uses a threshold-based segmentation method, the method presented here is technically independent of the segmentation method used. Future work will focus on identifying and adapting other methods to allow greater flexibility in data segmentation. Use of the Rayburst Sampling algorithm optimizes accuracy in quantifying 3D spine morphology, by avoiding residual optical image smear that can distort spine shapes, and by minimizing quantization error that limits the accuracy of digitized images. Most importantly, the ability to operate in 3D is a fundamental requirement of a spine analysis tool. As demonstrated in Figure 7, existing 2D detection methods can substantially underestimate spine counts [45], misrepresenting spine densities in morphometric studies. Nor can spine morphologies, volumes or surface areas, essential parameters in biophysical models that relate neuronal firing patterns to their structure [46]–[48], be quantified accurately with 2D methods.

Extending the method to different imaging modalities is a direction for further research. Live neuron imaging requires the ability to work in significantly lower axial resolutions, with highly asymmetrical voxel dimensions. Our future research will focus on evaluating the performance of the method under such conditions and developing new techniques to allow proper detection and classification of spines in live imaging. The automated spine analysis algorithms presented in this study provide a much-needed tool for the objective evaluation of morphometric changes that occur with synaptic plasticity, normal development and aging, and with neurodegenerative disorders that impair normal cognitive function.

## Materials and Methods

### Animals and Data Acquisition and Preprocessing

Four 9 month old male mice (C57Bl/SJL) were used. Animals were anesthetized with choral hydrate (15% aqueous solution, i.p.) and were perfused transcardially with 4% paraformaldehyde and 0.125% glutaraldehyde in phosphate buffer saline (PBS; pH 7.4). The brains were then carefully removed from the skull and postfixed for 6 hours. All procedures were conducted in accordance with the National Institute of Health Guide for the Care and Use of Laboratory Animals and were approved by the Mount Sinai School of Medicine Institutional Animal Care and Uses Committee.

For intracellular injections, brains were coronally sectioned at 200 µm on a Vibratome (Leica, Nussloch, Germany). The sections were then incubated in 4,6-diamidino-2-phenylindole (DAPI; Sigma, St. Louis, MO, USA), a fluorescent nucleic acid stain, for 5 minutes, mounted on nitrocellulose filter paper and immersed in PBS. Using DAPI as a staining guide, individual layer II/III pyramidal neurons of the frontal cortex were loaded with 5% Lucifer Yellow (Molecular Probes, Eugene, OR, USA) in distilled water under a DC current of 3–8 nA for 10 minutes, or until the dye had filled distal processes and no further loading was observed [45], [49]. Tissue slices were then mounted and coverslipped in Permafluor. Dendritic segment and spine imaging was performed using a Zeiss 410 confocal laser scanning microscope (Zeiss, Thornwood, NY, USA) using a 488 nm excitation wavelength, using a 1.4 N.A. Plan-Apochromat 100× objective with a working distance of 170 µm and a 5× digital zoom. After gain and offset settings were optimized, segments were digitally imaged at 0.1 µm increments, along the optical axis. The confocal stacks were then deconvolved with AutoDeblur (MediaCybernetics, Bethesda, MD, USA).

*Supporting Information is available online* (Box S1)

## Supporting Information

Box S1 Pseudo-Code for Spine Cluster Building Algorithm
(0.02 MB DOC)
