# Supplementary material for: Automated Three-Dimensional Detection and Shape Classification of Dendritic Spines from Fluorescence Microscopy Images
Source: PLoS One. 2008 Apr 23;3(4):e1997. doi: 10.1371/journal.pone.0001997 (PMC2292261; doi:10.1371/journal.pone.0001997)
Supplement: Box S1 — Pseudo-Code for Spine Cluster Building Algorithm (0.02 MB DOC) [file pone.0001997.s001.doc]

Box S1: Pseudo-Code for Spine Cluster Building Algorithm

## BOX S1 CLUSTER BUILDING ALGORITHM

Definitions:

Algorithm operates on object voxels only (intensity value at or above threshold)

*DTS* is defined as the distance to the surface of the closest dendritic segment

Neighbor voxels are defined based on 26-connectivity (6 faces, 12 edges, and 8 corners)

MAX_SPINE_WIDTH = user specified maximum allowable spine width

TOLERANCE = (smallest voxel dimension)/20

Loop for every exterior maximum

{

Create voxel containers CLUSTER, TEMP_I, and TEMP_J

Set PREV_LAYER to invalid

**loop**

{

Create voxel container CURR_LAYER

Set FLOOR to -infinity

Set spread of CURR_LAYER to zero

**if** PREV_LAYER is invalid

Insert maxima into TEMP_I and CURR_LAYER

**else**

Insert all voxels of PREV_LAYER into TEMP_I

**loop**

{

Insert all neighbors of TEMP_I into TEMP_J that are not in TEMP_I,

TEMP_J, any layer, any previous cluster, and have a *DTS* >= FLOOR

Clear TEMP_I

**if** TEMP_J is empty

**break**

**if** FLOOR is -infinity

Set FLOOR as the minimum *DTS* of voxels in TEMP_J minus TOLERANCE

Add voxels of TEMP_J to CURR_LAYER

Move voxels from TEMP_J to TEMP_I

Compute spread of CURR_LAYER

**if** spread of CURR_LAYER > MAX_SPINE_WIDTH or CURR_LAYER touched

the edge of the dataset

{

Set spread of CURR_LAYER to infinity

**break**

}

}

Append CURR_LAYER to CLUSTER

**if** CURR_LAYER is empty or spread of CURR_LAYER is infinity

**break**

Set PREV_LAYER to CURR_LAYER

}

Save CLUSTER

}
